# Cardiomyopathies and myocardial disorders in Africa: present status and the way forward

**DOI:** 10.5830/CVJA-2012-046

**Published:** 2012-11

**Authors:** Ayodele O Falase, Okechukwu S Ogah

**Affiliations:** Division of Cardiovascular Medicine, Department of Medicine, University College Hospital, Ibadan, Oyo State, Nigeria; Division of Cardiovascular Medicine, Department of Medicine, University College Hospital, Ibadan, Oyo State, Nigeria

**Keywords:** cardiomyopathy, Africa, sub-Saharan Africa

## Abstract

**Abstract:**

A review of heart diseases in Africa shows that the cardiomyopathies continue to be important causes of morbidity and mortality in the population. Hypertension remains the commonest cause of myocardial disease, followed by the cardiomyopathies. Ischaemic heart disease continues to be rare. Of the cardiomyopathies, dilated cardiomyopathy (DCM) is still the commonest. A large proportion of patients diagnosed with DCM in Africa have been shown to be cases of hypertensive heart failure, with varying degrees of myocardial dysfunction. Hypertrophic cardiomyopathy, which in the past was thought to be rare among Africans, has been shown to have the same prevalence as in other parts of the world. Moreover it is now known to be a genetic disorder. Endomyocardial fibrosis has become rare in communities where it used to be common. Its aetiology continues to be elusive. Arrhythmogenic right ventricular cardiomyopathy has been reported among Africans but there are no reports of left ventricular non-compaction or the ion channelopathies from Africa. Lenegre disease and the long-QT syndromes are well-known entities in clinical practice in Africa although long-QT in Africa is associated with potassium deficiency arising from prolonged treatment with diuretics. Left ventricular non-ischaemic aneurysms still occur but are rare. In view of these, a new classification of myocardial disorders was proposed for Africa.

## Abstract

For the past 60 years, reports from all parts of Africa have shown a remarkable unanimity in the pattern of heart diseases among black Africans.^1^,^2^ While ischaemic heart disease is the dominant cardiovascular disease in the Western world, it is rare in black Africans.

The typical pattern of heart failure in the sixties in a black African hospital is shown in Fig. 1A.^1^ From this figure, it is obvious that the commonest cause of heart failure was hypertension. This was followed by diseases that primarily affected the myocardium (labelled myocardial disease in the illustration), then rheumatic heart disease, followed by endomyocardial fibrosis. Recent data (Fig. 1B) among the same ethnic group show that the prevalence of hypertension has doubled, there is no difference in the prevalence of dilated cardiomyopathy, the prevalence of ischaemic heart disease remains low, while that of rheumatic heart disease has decreased considerably.^3^

**Fig. 1:**
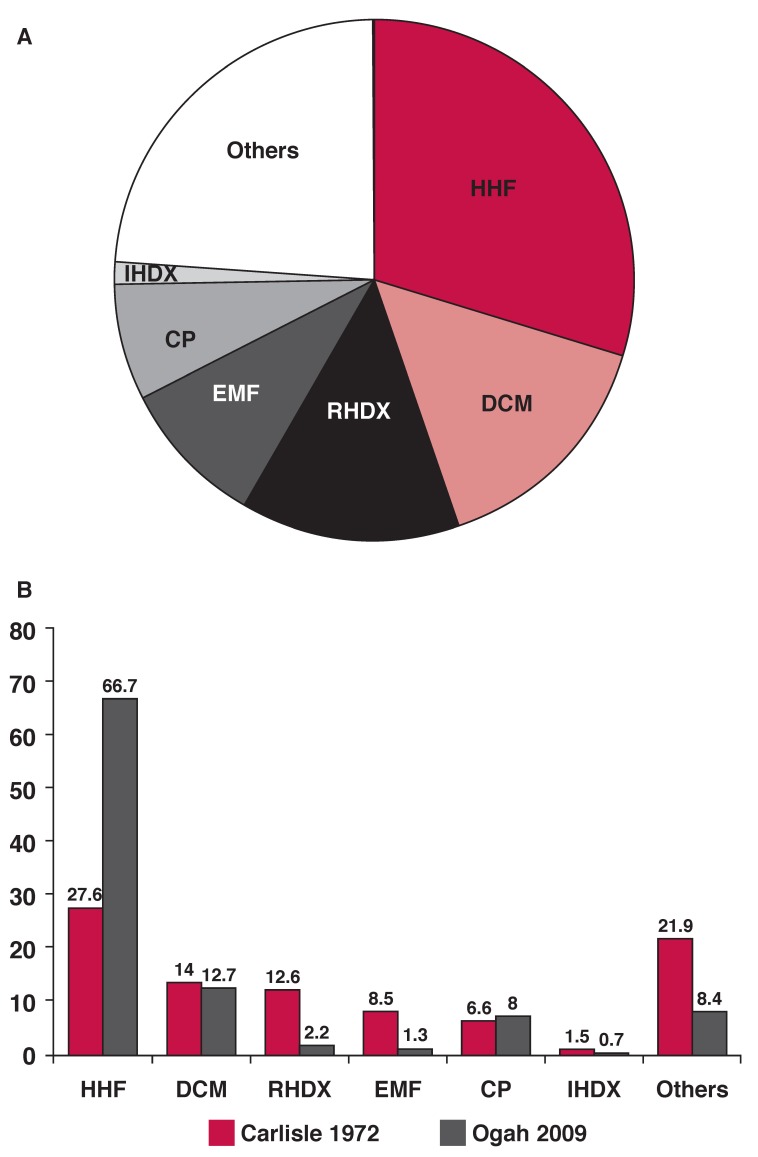
Causes of heart failure in Nigerians at the University College Hospital, Ibadan between 1968 and 1969 (Carlisle and Ogunlesi 1972). B. Causes of heart failure in the same ethnic group in the year 2010 compared with the 1972 data.^3^ HHF = hypertensive heart failure; RHDX = rheumatic heart disease; DCM = dilated cardiomyopathy; EMF = endomyocardial fibrosis; CP = cor pulmonale; IHDX = ischaemic heart disease.

We are primarily concerned with myocardial diseases and endomyocardial fibrosis in this discourse. We performed a systematic search of Pubmed for published data on cardiomyopathies in sub-Saharan Africa from January 1960 to December 2009. This was supplemented with parallel searches of references of identified journals, as well as a search of specific data sets such as African Index Medicus, African Journals Online (AJOL) and the World Bank database. The search strategy was Africa or sub-Saharan Africa and cardiomyopathy, dilated cardiomyopathy, endomyocardial fibrosis, and peripartum cardiomyopathy.

Prior to 1980 there were several descriptions from many geographical areas of the world but mainly from Africa of obscure forms of heart disease that primarily affected the heart muscle. These descriptions were published under several names such as idiopathic cardiomegaly,^4^ nutritional heart disease,^5^ cardiovascular collagenosis with parietal endocardial thrombosis,^6^ and cardiomyopathy.^7^ The common features of these descriptions were that affected patients presented in congestive cardiac failure with cardiomegaly, the cause of which was not readily apparent. The disease was particularly common in the tropical and sub-tropical countries of the world where it constituted one of the major clinical and health problems.^8^

Similarly, there were several descriptions from many parts of the world, of another group of myocardial diseases that was characterised by inappropriate massive hypertrophy of the cardiac muscle (HCM). It was first described by Teare in 1958.^9^ Prior to 1980, it appeared to have a worldwide distribution although initial reports suggested that it was rare in black people. The reason for this might have been due to a high prevalence of hypertensive heart disease, which interfered with the correct diagnosis of the disease.^10^ HCM too had been described in the past under several names, including idiopathic hypertrophic sub-aortic stenosis, muscular sub-aortic stenosis, obstructive cardiomyopathy and asymmetrical hypertrophy.^11^

Thirdly, there were before 1980 several descriptions of another group of myocardial disorders of unknown origin, which was characterised by fibrosis of the endomyocardium, particularly the inflow tract, apex and part of the outflow tract of either or both ventricles. The mitral/tricuspid valve apparatus, depending on which ventricular chamber was affected was commonly enmeshed in fibrous tissue, which very often involved the posterior valve leaflet. The anterior valve leaflet was rarely involved. It was called endomyocardial fibrosis (EMF) in those tropical countries that first reported it.^12^

Prior to the description of the tropical forms of EMF, there were also reports of a similar disease that was characterised by the presence of hypereosinophilia. It was first described by Löffler in 1936,^13^ and was known as Löffler’s endocarditis parietalis fibroplastica. This disease was initially believed to be confined to only the temperate zones of the world and was considered to be a separate illness from the tropical forms of EMF. However several reports have now confirmed its presence in Africa and other countries of the world where the tropical forms of EMF is prevalent.^14^ Some have even suggested that it is the early form of tropical EMF (Fig. 2).^14^

**Fig. 2:**
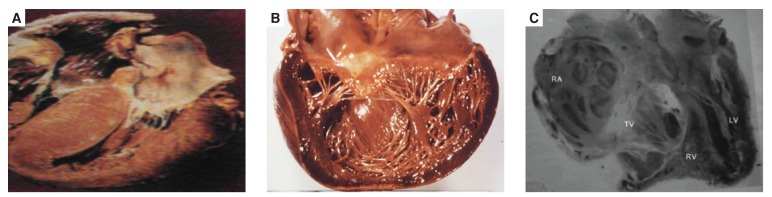
Hypertrophic cardiomyopathy; B. dilated cardiomyopathy; C. endomyocardial fibrosis.

## Classification of the cardiomyopathies

Because of the initial confusion arising from the names given to all these diseases, the inadequacy of previous classifications,^15^ and to ensure unanimity in their descriptions, a task force was set up by the World Health Organisation and the International Society and Federation of Cardiology (WHO/ISFC) to harmonise the features of the diseases, adopt uniform names for them, and design an acceptable classification. The report of the task force was published in 1980,^11^ while the full description of each disease was published by the WHO as a report of an expert committee in 1984.^10^

At the 1980 meeting, it was agreed to adopt the name ‘cardiomyopathy’ for all diseases of the heart muscle of unknown cause. For a disease to qualify as a cardiomyopathy therefore, the patient must have been extensively investigated and no cause found for the malady. Diseases whose causes were known were simply identified by their causative factors, such as alcohol heart disease, myocarditis or viral heart disease. Such diseases were grouped under a separate heading called ‘specific heart muscle diseases’ which was defined as ‘heart muscle diseases of known cause or associated with disorders of other systems’.

Since it was considered inappropriate to slot well-known disorders of the myocardium caused by systemic or pulmonary hypertension, coronary artery disease and congenital heart diseases under this group, they were excluded from the classification. It was felt that all diseases of the myocardium would have had to be listed under specific heart muscle diseases if this was not done. In summary, cardiomyopathy was regarded as a disease of exclusion.

The meeting further agreed to group the cardiomyopathies into three types. The first two were named to highlight the changes in their structures (hypertrophic and dilated) while the third, unfortunately, in our view, was named to reflect its restrictive haemodynamic features (restrictive). This classification was made before arrhythmogenic right ventricular dysplasia was characterised and identified as a separate disease in the literature, hence its omission in the classification.

The reaction of clinicians/researchers to this classification was mixed. While everyone agreed with the designation of these diseases as cardiomyopathies, not everyone agreed with its classification. Several classifications followed thereafter but, in so doing, many of them again extended the definition of cardiomyopathies to include any disorder of the myocardium.

The report of another task force, set up by the WHO in 1995^16^ to update the 1980 classification, defined the cardiomyopathies as any disease of the myocardium with cardiac dysfunction, although it retained the original three types of cardiomyopathies: hypertrophic, dilated and restrictive. It added a fourth one, arrhythmogenic right ventricular cardiomyopathy (formerly known as arrhythmogenic right ventricular dysplasia). But while it recognised that dilated cardiomyopathy may derive its origin from conditions such as viral myocarditis, it created, under specific cardiomyopathies, an entity known as inflammatory cardiomyopathy, which it defined as ‘myocarditis in association with cardiac dysfunction’.

Similarly it also created another entity, known as alcoholic cardiomyopathy, under specific cardiomyopathies after it had agreed that dilated cardiomyopathy may be caused by the consumption of alcohol. Moreover, ischaemic cardiomyopathy was recognised in the report as a separate entity under specific cardiomyopathies, although the same disease was earlier said to be a variety of dilated cardiomyopathy. Finally, the document introduced new names for well-recognised diseases. For example, hypertensive cardiomyopathy was substituted for the well-known and more specific terms, hypertensive heart disease/hypertensive heart failure.

The latest classification, sponsored by the American Heart Association,^17^ agreed to classify the cardiomyopathies in line with ‘the molecular era of cardiovascular disease’. It also agreed that cardiomyopathies should include any disorder of the myocardium and therefore defined them as ‘a heterogeneous group of diseases of the myocardium associated with mechanical and/or electrical dysfunction that usually (but not invariably) exhibit inappropriate ventricular hypertrophy or dilatation and are due to a variety of causes that are frequently genetic. Cardiomyopathies either are confined to the heart or are part of generalised systemic disorders, often leading to cardiovascular death or progressive heart failure-related disability’.

The panel made a case for inclusion of the ion channelopathies in the classification of the cardiomyopathies, although it agreed that they are primary electrical diseases with no gross or histopathological abnormalities. As in the 1980 classification, it excluded from the list of cardiomyopathies ‘pathological myocardial processes and dysfunction that are a direct consequence of other cardiovascular abnormalities, such as that which occurs with valvular heart disease, systemic hypertension, congenital heart disease, and atherosclerotic coronary artery disease producing damage secondary to impairment in coronary flow’.

The report went further to classify the cardiomyopathies into two major groups ‘based on predominant organ involvement’. These were primary and secondary cardiomyopathies. Primary cardiomyopathies, which may be genetic, non-genetic or acquired, were defined as ‘those solely or predominantly confined to heart muscle and are relatively few in number’. The genetic forms included hypertrophic cardiomyopathy, arrhythmogenic right ventricular cardiomyopathy (ARVC), left ventricular non-compaction, conduction system disease, glycogen storage disorders, mitochondrial myopathies, and ion-channel disorders. The mixed forms were dilated cardiomyopathies and the restrictive (non-hypertrophied and non-dilated) types.

In the report, secondary cardiomyopathies were diseases that were associated with systemic illnesses. These were similar to those listed under specific heart muscle disease and specific cardiomyopathies by the 1980 and 1996 reports, respectively.

## Classification of the cardiomyopathies in Africa

For clinicians working in Africa where the cardiomyopathies are most prevalent, the question that follows is which of these classifications is appropriate for Africa? Before we answer this question, let us briefly review what is currently known about the cardiomyopathies in Africa.

## Hypertrophic cardiomyopathy

In the pre-echocardiography era, hypertrophic cardiomyopathy (HCM) was hardly diagnosed in Africa. All abnormal electrocardiographs (ECGs) suggestive of left ventricular hypertrophy (LVH) were attributed by clinicians to hypertensive heart disease since hypertension was the dominant cardiovascular disease among Africans and was extremely common in African populations. The widespread use of echocardiographic examination all over Africa changed all this as it showed that HCM indeed occurs among Africans.

For instance, HCM was found in 0.2% of 6 680 unselected echocardiograms in Tanzania^18^ and in 2% of 712 echocardiograms in Lagos, Nigeria.^19^ Similar reports have also come from South Africa where extensively genetic investigations have confirmed similar findings with other parts of the world.^20^ In Ghana,^21^ 1.15% of 572 patients referred for echocardiography at the Ghana National Cardiac reference centre had HCM, while Abegaz in Ethiopia discovered that 53 out of 1 240 abnormal echocardiograms performed at the Armed Forces General Hospital had HCM.^22^ The prevalence rate among populations outside Africa ranged from 0.2 to 0.5%. Interestingly, Maron *et al.*^17^ found in their CARDIA study that the prevalence of HCM in blacks was twice that of whites, while more African-Americans performing competitive sports died suddenly compared with their white counterparts.

The aetiology of HCM has similarly been sorted out. Investigations all over the world have conclusively shown that HCM is inherited as an autosomal dominant genetic disorder that is caused by mutations in at least 10 different genes that code for sarcomeric proteins.^2^,^15^ Mutations in the β-myosin heavy chain gene, myosin-binding protein C and troponin T account for 70 to 80% of all the cases. The total number of mutations is well over 100, and new mutations are being discovered. HCM is therefore no longer a heart muscle disease of unknown cause and this implies that the 1980 report on the cardiomyopathies is no longer valid.

## Arrythmogenic right ventricular cardiomyopathy (ARVC)

Reports from Africa about this disease have come from only South Africa.^20^,^23^ There are familial cases of ARVC as well as non-familial ones. Although the disease has been associated with enteroviral and adenoviral myocarditis, it is not considered to be primarily caused by myocarditis. Lately it has been shown that the disease is not confined to the right ventricle as the name suggests, because the left ventricle may be affected in up to 75% of the patients.^12^

## Other genetic disorders of the myocardium

Familial and non-familial cases have been described in patients with the recently discovered myocardial disorder known as left ventricular non-compaction (LV non-compaction).^12^ Characteristic morphological changes of this disease are often found in the left ventricle of those with the disease. There are no reports of LV non-compaction from Africa, possibly because African cardiologists are not yet familiar with its echocardiographic changes.

Genetic disorders of the electrical system of the myocardium with or without morphological changes have also been described. They include Lenegre disease,^17^ a progressive disease of the conduction system of the heart and the ion channelopathies. Among the ion channelopathies are the long-QT syndrome, Brugada syndrome, catecholaminergic polymorphic ventricular tachycardia, short-QT syndrome and idiopathic ventricular fibrillation, all of which can cause sudden death. Lenegre disease and the long-QT syndromes are well-known entities in clinical practice in Africa although long-QT is only associated in Africa with metabolic problems, particularly potassium deficiency following prolonged treatment with diuretics. It is however interesting to note the recent report from South Africa,^24^ which concluded that the genetic forms of long-QT syndrome (LQTS) occurred most commonly among the white Caucasian population of South Africa, with fewer cases from those of mixed ancestry and none from those of black African descent.^2^ The other ion channelopathies have not been reported from Africa, probably because of lack of sophisticated cardiac electrophysiological studies and a dearth of well-trained personnel required to make the diagnosis.

## Dilated cardiomyopathy

Next to hypertension, this is the commonest cause of heart failure in black Africans.^1^,^2^ In some communities in Africa, DCM is the commonest cause of heart failure. Before the advent of echocardiography, the diagnosis was made on the basis of clinical presentation, chest X-ray, ECG, and sometimes in the large teaching hospitals, cardiac catheterisation and angiography. Echocardiography has made the diagnosis easier and is presently the preferred investigation for making a diagnosis.

Affected patients often presented in congestive cardiac failure with functional mitral and tricuspid regurgitation due to myocardial failure, the cause of which was not apparent. ECG changes were variable. Some had low-voltage complexes while others presented with left ventricular hypertrophy. Abnormal intraventricular conduction defects were common, especially left bundle branch block. Chest X-ray usually showed cardiomegaly with failure, while angiography confirmed a dilated left ventricle with poor myocardial contraction and functional mitral regurgitation.

DCM is widely regarded as an end-stage myocardial disease from a wide variety of adverse factors. The most common of these insults are briefly considered below.

## Hypertension

For a long time, several workers in Africa had suspected that many patients who were labelled as having DCM were really hypertensives. And there had been several debates at world conferences where this assertion was actively advanced by many workers. Mokhobo,^25^ from South Africa advised caution in making a diagnosis of cardiomyopathy since ‘cardiomyopathy and hypertension are both common in black patients and confusion may arise between them’. Lowenthal,^24^ also from South Africa wrote as follows: ‘these cases are regarded as evidence in favour of the hypothesis that many cases of cryptogenic heart disease (cardiomyopathy, congestive cardiomyopathy, idiopathic cardiomegaly) are in fact hypertensives presenting with normotensive cardiac failure’.

From Nigeria, Brockington,^26^ after extensive studies, came to the conclusion that hypertension and what he called heart muscle disease at the time were similar and that the latter was ‘the late stage of untreated chronic hypertensive heart failure’. Brockington’s view was supported by Celia Oakley at a debate during a conference on cardiomyopathies held in London in 1971 but John Goodwin at the same conference disagreed, asserting that hypertensive heart disease was structurally different from heart muscle disease.

The contentious problem in the past had always been the presenting blood pressure. Some patients had a normal blood pressure at presentation but in others the blood pressure was mildly raised, although out of proportion with the degree of the patient’s heart failure. Very often, the hypertension was transient, the blood pressure becoming normal with treatment of the heart failure and staying normal without sustained treatment with diuretics or hypotensive agents. Some investigators had referred to this phenomenon as ‘reactive hypertension’ or ‘Sahli’s Hochdruckstauung’^27^ because it was thought to be due to intense peripheral vasoconstriction, which occurred in heart failure.

**Fig. 3:**
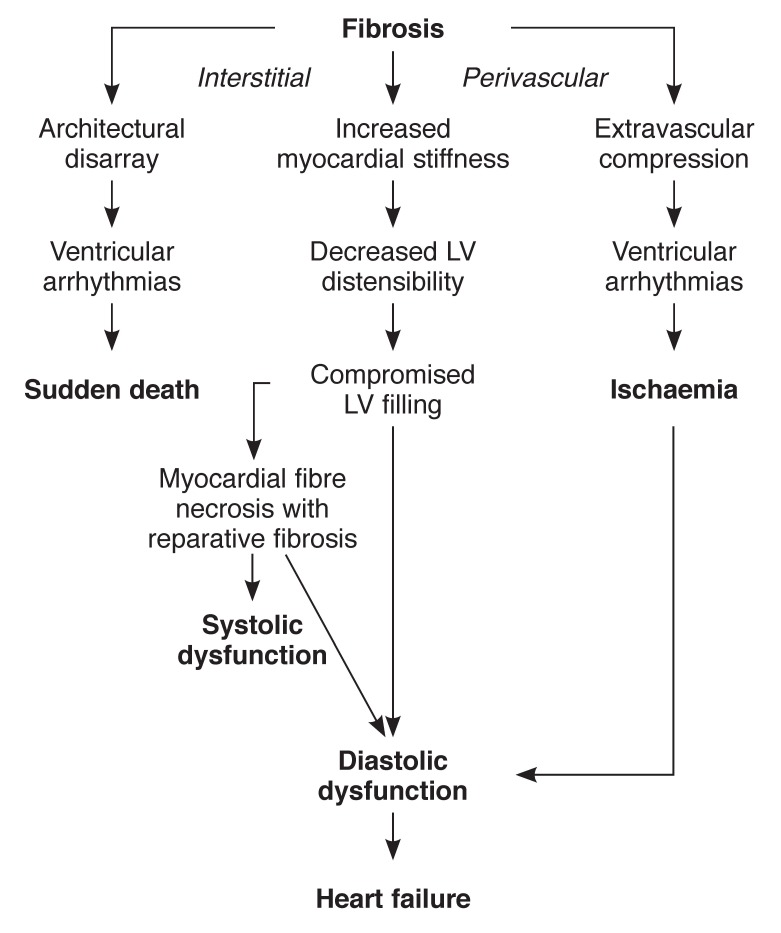
The consequences of myocardial fibrosis on the heart of patients with arterial hypertension, modified from Diez *et al. Nature Clin Pract Cardiovasc Med* 2005; 2(4): 209–216.

**Fig. 4:**
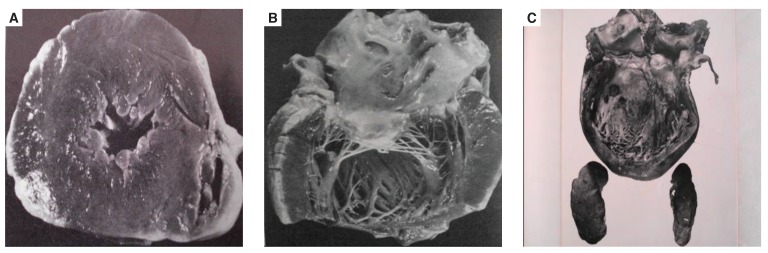
Representation of progression of the left ventricle in hypertensive patients, from concentric hypertrophy with a small cavity, to concentric hypertrophy with a large cavity, to a destroyed myocardium unable to sustain a high blood pressure.

Studies from Nigeria in the seventies have however shown that what was called heart muscle disease in the past was not caused by a single disease process.^28^-^33^ Over 75% of patients were hypertensives whose hearts had become damaged over time because of poor or lack of control of high blood pressure.^34^ Progression from a hypertrophied heart (concentric or asymmetric) in Nigerian hypertensives to the stage of flabby heart has been discussed in an earlier publication (Figs 3, 4).^34^

## Alcohol

Early reports from Nigeria showed that alcohol consumption played a significant part in the genesis of myocardial damage of patients diagnosed with cardiomyopathies. The authors also suggested that excessive consumption contributed to the heart failure of some of their hypertensives.^29^,^35^ This is in agreement with Rees *et al*.^36^ who in 1974 suggested from Nairobi that some of their patients with cardiomyopathies were suffering from the combined effects of excessive alcohol consumption and hypertension, and this combination led to congestive cardiac failure.

Some of the patients studied in Nigeria had thiamine deficiency and these were linked with protein malnutrition and excess consumption of alcohol. All but one had low-output cardiac failure and they did not respond to thiamine administration.^30^ By contrast, high-output and low-output cardiac failure caused by thiamine deficiency was shown to have equal prevalence in South African alcoholics.^27^

Generally, it is now estimated that alcohol is a contributory factor in a significant number (up to 45%) of patients with heart failure of unknown cause in Africa,^27^ and alcohol also contributes to the heart failure of a significant number of hypertensives.

## Myocarditis

Studies in the seventies also showed that myocarditis was the cause of a significant number of patients diagnosed at the time as heart muscle disease of unknown cause, especially among young people below the age of 30 years. At that time, it was only possible to investigate the role of Coxsackie B virus and *Toxoplasma gondii* in the patients studied, but higher antibody levels were found in the patients compared with control subjects. About 45% of the patients eventually turned out to be hypertensive, implying that myocarditis was playing some part in the genesis of their myocardial damage.^28^-^30^,^32^

A few cases of acute myocarditis caused by Coxsackie B_3_ virus and *Toxoplasma gondii* were documented during the four years the patients were followed up. Sub-clinical infection by *Toxoplasma gondii* is common in Nigeria and several studies have found that virtually everyone living in this community has seroconverted to the organism.^32^,^33^

Since then, several studies have confirmed the role of myocarditis in the genesis of myocardial failure all over the world and many more organisms [viruses including the human immunodeficiency virus (HIV), bacteria including mycobacteria, parasites such as *Trypanosoma, Toxoplasma gondii* and *Schistostoma*] have been identified as culprits.^2^ In Africa, many patients with myocardial failure and a positive HIV test have been shown to have not only viral myocarditis (HIV, Epstein-Barr virus, cytomegalovirus, parvovirus, adenovirus and human simplex virus) but also infections from other organisms such as toxoplasmosis and cryptococcus.

Endomyocardial biopsies and detection of viral genomes have been crucial in the identification of these agents in the myocardium of the patient.^37^,^38^ In an excellent study by Shaboodien,^39^ a prevalence of 100% infectivity (enterovirus, Epstein-Barr virus, parvovirus, human simplex virus and adenovirus) was found in the hearts of the patients with ‘idiopathic dilated cardiomyopathy’ she biopsied. Unfortunately these advanced techniques are not widely available in Africa outside South Africa.

The role of excessive immune activation in the pathogenesis of the disease has also been studied in Africa. A study found that HLA-DR1 and HLA-DRw10 were commoner in patients with DCM.^40^ Elevated plasma levels of inflammatory cytokine tumour necrosis factor (TNF)-α, C-reactive protein and a plasma marker of apoptosis have also been found in DCM patients,^41^-^43^ and those with peripartum cardiomyopathy.^44^ Leucocyte cytokines were also found to be elevated in these patients. In those with peripartum cardiomyopathy, the level of Fas/Apo-1 was also elevated.

These findings had led to studies on the immunomodulatory effect of pentoxifylline in the management of the DCM patients, with promising results.^45^ The process of progression from acute myocarditis to dilated cardiomyopathy was recently elucidated by Kawai.^46^

## Peripartum cardiomyopathy (PCM)

PCM commonly occurs during the last trimester of pregnancy and the first six months after delivery. Risk factors that have been reported in the literature are:^37^,^47^-^50^

• black race
• advanced maternal age
• twin pregnancies
• gestational hypertension
• long-term tocolysis.

Reports from Haiti have shown that partial improvement of systolic function occurred in the first year of follow up.^50^ In fact a third of their women with PCM recovered fully within five years. Mortality rates were 15% in the first six months and 42% during 25 years of follow up. The Hausas in the northern part of Nigeria have the highest known incidence of PCM in the world – about 13% of all female admissions at the Ahmadu Bello University Teaching Hospital, Zaria.^51^-^54^

A wide range of diseases has been advanced as the cause of myocardial damage of these women. They include myocarditis, hypertension, abnormal immune response to pregnancy, impaired cardiac (systemic) microvasculature, increased myocyte apoptosis, cytokine-mediated inflammation, selenium deficiency (Keshan disease), lying on heated mud beds after taking two hot baths in addition to excessive consumption of a special porridge to which as much as 30 g/day of potash (kanwa) has been added (Zaria cases), genetic predisposition, increased adrenergic tone leading to myocardial stunning, and excessive production of prolactin.^55^

Recent studies have also shown that unbalanced peri/post partum is linked to proteolytic cleavage of prolactin, which turns it into a potent anti-angiogenic, pro-apoptotic and pro-inflammatory factor. These observations tend to suggest that prolactin cleavage could operate as a specific pathomechanism for the development of PCM.^55^,^56^ Bromocriptine has been shown to be beneficial in reducing the raised levels of prolactin found in some of the women, improving their left ventricular function.^57^

The plasma marker of apoptosis, Fas/Apo-1 has also been shown to be elevated in patients with PCM,^42^,^45^ while troponin T levels have been found to be useful in predicting the presence of persistent left ventricular dysfunction in patients.^58^ Baseline Fas/Apo-1 levels and higher NHYA at presentation were found to be the only predictors of mortality, while MRI using late gadolinium enhancement can be useful in evaluating the extent of myocardial damage and predict the outcome of the disease.^46^,^59^,^60^

With molecular studies, viral genomic materials of enterovirus, parvovirus B_19_, human herpes virus 6, Epstein-Barr virus and cytomegalovirus have been identified in the heart muscle of some women with PCM.^61^-^63^ On the contrary, Cenac *et al*.^64^ found no evidence of viral myocarditis in Niger, West Africa. Sanderson *et al*.,^37^ O’Connell *et al*.^65^ and Midei *et al*.^66^ on the other hand found evidence of myocarditis in biopsy materials of the women they studied. In fact, Midei *et al*.^66^ reported that as many as 78% of newly diagnosed patients with PCM seen by his group had myocarditis and resolution was associated with improvement of left ventricular function.

## Familial dilated cardiomyopathy

Familial forms of dilated cardiomyopathies have been well summarised by Maron *et al*.^17^ It is estimated that they constitute 20 to 35% of cases of DCM. They are genetically heterogenous but the predominant mode of inheritance is autosomal dominant, with X-linked autosomal recessive and mitochondrial inheritance occurring less frequently. Many of the mutant genes that are linked to autosomal dominant DCM also encode the same contractile sarcomeric proteins that are responsible for hypertrophic cardiomyopathy.

According to Maron *et al*.,^17^ DCM is also caused by a number of mutations in other genes encoding cytoskeletal/sarcolemmal, nuclear envelope, sarcomere and transcriptional co-activator proteins. The most common of these is the lamin A/C gene, which is also associated with conduction system disease, and encodes a nuclear envelope intermediate filament protein.

In Africa, the first report of familial DCM was from Uganda where a set of twin brothers was reported to have had the disease.^67^ Reports of familial cases have also come from South Africa. A case of familial DCM with prominent ventricular arrhythmias was reported by Brink *et al*.^68^ while Przybojewski *et al*.^69^ described two brothers of Afrikaner ancestry with unexplained DCM. Cases of DCM have been linked with progressive familial heart block types I and II.^70^-^72^ There were also reports of DCM in two brothers,^68^ and a case of microcephaly associated with DCM.^72^

Mayosi *et al*.^73^ has also shown that actin mutations do not play a major role in DCM but a mitochondrial DNA susceptibility gene increases the risk of DCM in the general population. Other studies from South Africa evaluated the role of mutations in signaling pathway proteins, in particular, polymorphisms in the angiotensin-converting enzyme and beta-adrenoreceptor subtypes in the progression of DCM.^74^

## Malnutrition

Initial reports of DCM from South Africa suggested that the disease was caused by malnutrition. The patients whom Gillanders^5^ and Higginson *et al*.^75^,^76^ studied improved when their diet was changed to what was described as a ‘balanced’ one. This view has been discarded as Gillanders’ experiments could not be reproduced.

In our study, we found that patients with DCM had significantly lower serum albumin levels compared with controls, irrespective of whether they consumed alcohol or not. This was attributed to hepatic dysfunction caused by congestive cardiac failure.^29^

## Haemosiderosis

Consumption of iron in excess from iron-containing beer (Bantu haemosiderosis) has been suggested as a potentially reversible causative factor of DCM.^75^ It has also been found that the ‘poor correlation of cardiac and hepatic iron deposits with heart disease’ might have led to the under-recognition of dietary iron overload as an important factor in the pathogenesis of DCM.^77^ High levels of serum ferritin have been found in patients with DCM compared with patients with heart failure from other causes, and about 50% of the patients had ferritin levels above 500 ng/ml.^30^

## Hb genotype

No association was found between Hb genotype and DCM in the patients we studied and there are no reports linking different types of Hb genotypes with DCM.

## Summary of studies on DCM in Africa

From all these studies, we can conclude as follows:

• Hypertension contributes to some of the cases diagnosed clinically as DCM in Africa. Such patients should be identified and reclassified as having hypertensive heart failure with varying degrees of myocardial dysfunction.
• Chronic alcohol consumption and myocarditis have also been identified as the causes of myocardial damage in some of the patients clinically diagnosed as cardiomyopathy in Africa and even worldwide.
• Familial cases of DCM have been described in Africa but limited research has been done on the continent.
• Lack of facilities and technical know-how have also made the routine diagnosis of myocarditis in Africa impossible. Many are therefore missed.
• Estimation of markers of inflammation such as C-reactive protein, inflammatory markers such as the tumour necrosis factor α, and the plasma marker of apoptosis (Fas/Apo-1) may perhaps help in making a preliminary diagnosis of myocarditis. This needs to be further explored.
• In patients with PCM, abnormal immune response to pregnancy, increased myocyte apoptosis, selenium deficiency, cultural practices such as those of the Zaria women, and prolactin excess/cleavage are other causes that have been advanced by workers in the field. In those with excess prolactin production/cleavage, bromocriptine has been found to improve the cardiac status of these women.
• Thiamine deficiency is common among Africans with DCM and it is related to excessive alcohol consumption and/or malnutrition. By itself, it is not an important cause of DCM.
• Haemosiderosis has been found in many patients with DCM and is probably due to excessive consumption of alcohol stored in iron containers. Haemosiderosis is reversible.

## Implication

For a patient’s heart failure to qualify to be labelled as dilated cardiomyopathy according to the 1980 classification of the cardiomyopathies,^11^ a patient must have been extensively investigated and no cause for his/her disease found. Under this classification, diseases whose causes are known should be named appropriately.

From the above, therefore, we can assert that some of the patients diagnosed with DCM in Africa have hypertensive heart disease/failure. Others have alcohol heart disease, viral heart disease or myocarditis, peripartal heart disease and familial dilated heart muscle disease. There are other cases of DCM whose aetiology does not fall into these categories. The prevalence of such cases varies among the different communities of Africa.

## Restrictive cardiomyopathy

Under the 1980 classification,^11^ the term restrictive cardiomyopathy was in our view out of place. While hypertrophic and dilated cardiomyopathies referred to structural changes within the heart, restrictive cardiomyopathy referred to haemodynamic changes. It would have been more consistent if a term such as fibrotic/obliterative cardiomyopathy was used to describe endomyocardial fibrosis (EMF) and Löeffler’s endomyocardial disease, the two diseases the report had in mind. Moreover, with the advent of echocardiography, it is now known that diastolic problems of the heart are not limited to endomyocardial fibrosis and its variants. They occur in several other diseases, including hypertension and hypertrophic cardiomyopathy, and indeed in infiltrative disorders such as amyloidosis.

Under the 1980 classification,^11^ it was recommended that Löffler’s cardiomyopathy be relabelled eosinophilic endomyocardial disease (EED). Both EED and EMF are characterised by scarring of the endomyocardium of either or both ventricles, which creates cavity obliteration and restriction of filling of the ventricles with blood. While the outflow tract of the affected ventricle is spared, the atrio-ventricular valve becomes enmeshed with scar tissue, which consequently binds down the posterior valve leaflet and leaves the valve perpetually open. Free regurgitation of blood occurs because of this, resulting in an enormously dilated atrium.

EED was first described in Europe by Löffler^13^ in 1936, while EMF was first described by Bedford and Konstam^12^ in 1946 among Nigerian soldiers who served in the second world war. EMF was similarly reported by Davies^78^ in East Africa in 1947, although observations had been made about the disease by Arthur Williams^79^ as far back as 1938. Despite a myriad publications on the two diseases in the world literature, there is still no agreement on their aetiology.

In an excellent review, Bukhman *et al*.^80^ summarised the causes of EMF, which had been proposed by several authors in the literature as follows:

• infection – toxoplasmosis, rheumatic fever, malaria, helminth parasites
• allergy – eosinophilia, auto-immunity
• malnutrition – protein deficiency, magnesium deficiency
• toxic agents – cerium, cassava, thorium, serotonin, plant toxin, vitamin D.

In addition to these, the following were considered as probable causes from Ibadan, Nigeria:

• vitamin E (tocopherol) deficiency
• obstruction of cardiac lymphatics
• *Schistosoma* infestation: *Schistosoma* ova had been found in EMF lesions of some Nigerian patients.

Of all these, only the hypereosinophilic syndrome of Löffler has been shown to be definitely associated with fibrosis and obliteration of the cardiac apex, similar to tropical EMF. The cause of tropical EMF still remains largely unknown. However Löffler’s endomyocardial disease and the tropical forms of EMF, although similar, do not appear to be the same disease, as there are important differences between them. These can be summarised as shown in Table 1.

**Table 1.** Differences Between Tropical EMF And EED

*Parameter*	*Tropical EMF*	*EED (Loeffler’s)*
Constitutional symptoms	Absent	Present
Hypereosinophilic syndrome	Absent	Present
Degranulated and vacuolated eosinophils	Few cases	Invariably present
Cationic proteins	Not elevated	Elevated
Location of lesions	RV, LV or BV	Invariably BV
Geographical distribution	Mainly rainforest regions	Worldwide
Age group	Usually children	No specific age group
Eosinophilic infiltration of other organs	Absent	Present

EMF = endomyocardial fibrosis; EED = eosinophilic endomyocardial disease; RV = right ventricular; LV = left ventricular; BV = biventricular.

The questions that arise from all these studies are as follows:

1. What causes eosinophils to degranulate and attack the patient’s own tissues? In our view this has not been adequately addressed. It is known that eosinophils are normally deployed by the body to fight parasites and are often elevated in those with allergies, including drug reactions, collagen disorders such as polyarteritis nodosa and some malignancies. But these eosinophils do not harm the patient’s tissues. What therefore programmes/induces them to attack the patient’s tissues and organs and cause the fibrotic reactions seen in the hypereosinophilia of Löffler’s disease?
2. Can filaria worms induce eosinophils to proliferate on a massive scale (i.e. hypereosinophilia), infiltrate major organs of the body, degranulate and attack patients’ tissues and organs during the process of eliminating the invading organisms?
3. Are there other agents – toxins, infections such as *Toxoplasma gondii* and *Schistosoma mansoni* – that are capable of damaging the endomyocardium like hypereosinophilia?
4. Can parasites such as filaria worms, ova of common parasites such as *Schistosoma*, or other organisms such as *Toxoplasma gondii* get caught within the endomyocardium, cause chronic inflammation and subsequently fibrotic reactions? These parasites are known to lodge in various organs of the body such as the liver and lungs, where they induce fibrotic reactions, and it is often forgotten that they can also lodge within the myocardium of the heart. There may be an eosinophilic reaction to the parasite in such a situation but this will be mild and transient, and not on the same scale as Löffler’s endomyocardial disease.
5. Is the peculiar location of the lesions in EMF and EED due to the mode of blood flow through the ventricles? Studies have shown that there is relative stasis of blood flow within the apices of the ventricles and for this reason most clots congregate at the apices of the ventricles.
6. Is there a consensus with the pathogenesis proposed by Olsen,^81^ of fibrotic lesions within the endomyocardium of patients with EMF/EED? His studies showed that the process of development of EMF/EED goes through the following phases:
- necrotic phase with active myocarditis, inflammatory infiltrates and eosinophils
- thrombotic phase with endocardial thickening, thrombosis and decrease in the number of inflammatory cells
- fibrotic phase involving the endocardium, replacement of tissues by collagen and superficial thrombosis.
7. What is the implication of the recent observations in Uganda by Freers *et al*.,^82^-^84^ which showed that fibrosis in patients with EMF is not confined to the endomyocardium but also occurs in other tissues such as the peritoneum, pleura, liver and pericardium. Does it conform to the parasitic theory put forward in (4) above?
8. Are there others markers specific for Löffler’s endomyocardial disease that could make the diagnosis of the disease easier in Africa?

Answers to these questions will help researchers in Africa make significant progress in finding the cause(s) of EMF. The greatest problem we have with the disease at present is that we do not know how the illness begins. Several investigators have described what they believe are the early illnesses of the disease but these are not convincing. Yet, it is during the early stages of the disease that we can find its cause and define its pathogenesis.

**Fig. 5:**
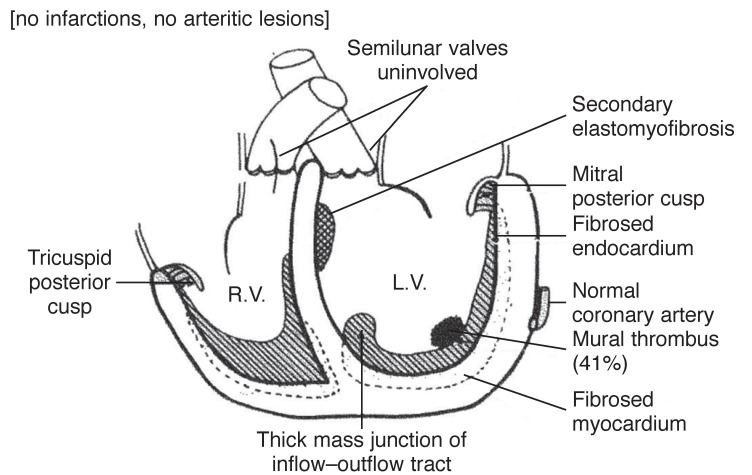
Davies’ representation of endomyocardial fibrosis.

The EMF cases seen in our wards in Africa are already in the chronic stages of the disease, when the cause is difficult or impossible to find. By going to the villages where there is poverty and a high incidence of EMF, and by using an echocardiogram to aid their study, the research team presently working in Mozambique^85^ has a unique opportunity to help us find the cause of the disease (Fig. 5).

## Left ventricular non-ischaemic ventricular aneurysms

These are ventricular aneurysms that occur in black people living in the tropics, particularly those who live in the equatorial rainforest belt of Africa, and are not due to or associated with coronary artery occlusion.^86^-^88^ They belong to the group of forgotten tropical cardiomyopathies. Although they are now rare, they still occur. Sporadic cases of such aneurysms have been described in the past from the United States of America, France, Sweden, South Africa and the West Indies, mainly among black people living in those countries. It was first reported in 1813 by Corvisart, in a black man who died of the disease in France in 1796.

The aneurysms are often sub-valvular in location, more commonly affecting the mitral and aortic valve rings. Sub-mitral aneurysms tend to be very large, often creating mitral regurgitation; sub-aortic aneurysms on the other hand tend to be smaller and may cause left and right ventricular outflow obstruction with aortic and pulmonary regurgitation. Sometimes the aneurysms are annular, extending in a circular direction around the heart and may extend laterally, superiorly and anteriorly. Left atrial aneurysms may be found in some patients.

Atrio-ventricular conduction abnormalities, including complete heart block are common. The cause of the aneurysms is unknown, although the pathological findings and the presence of fibrinous pericarditis support the theory that the out-pouchings are initiated by myocarditis.

## Proposed new classification

Earlier, we posed this question ‘which of the several classifications of myocardial disorders is suitable for Africa?’ The problem is that there is no agreement at present on the definition of cardiomyopathy. While some regard it as ‘any disorder of the myocardium’, others believe that it should be defined as heart muscle disease of unknown cause. Yet others believe that it should be defined as ‘any disease of the myocardium with cardiac dysfunction’. This last definition implies that patients with hypertensive heart disease/failure will be described as hypertensive cardiomyopathy and those with aortic valve disease for example, aortic valve cardiomyopathy.

While waiting for another consensus meeting to resolve this difficulty, we wish to propose the following classification (Table 2). It is our view that this could stimulate the continental society (PASCAR) to set up an expert committee to look into this subject.

**Table 2.** Proposed Classification Of Myocardial Disorders For Africa

**1. Diseases That Arise Within The Cardiovascular System**	**2. Diseases That Arise Outside The Cardiovascular System**
GENETIC	INFILTRATIVE
A. DISORDERS OF THE MYOCYTES	• Amyloidosis (primary, familial autosomal dominant, senile, secondary forms)
(i) Non-dilated types:	• Gaucher disease
• Hypertrophic cardiomyopathy	• Hurler’s disease
• LV non-compaction	• Hunter’s disease
(ii) Dilated types:	STORAGE
• Arrhythmogenic right ventricular cardiomyopathy	• Haemochromatosis
• Familial dilated cardiomyopathy	• Fabry’s disease
B. DISORDERS OFTHE ELECTRICAL STRUCTURES	• Glycogen storage disease (type II, Pompe)
(i) Conduction system disease	• Niemann-Pick disease
Structural types: Lenegre disease	TOXICITY
Non-structural types: ion channelopathies	• Drugs
• Long-QT syndrome	• Heavy metals
• Brugada syndrome	• Chemical agents
• Catecholaminergic polymorphic ventricular tachycardia	GRANULOMA
• Short-QT syndrome	• Sarcoidosis
• Idiopathic ventricular fibrillation	ENDOCRINE
NON-GENETIC	• Diabetes mellitus
(i) Hypertrophic	• Hyperthyroidism
• Hypertensive heart disease (concentric, asymmetric)	• Hypothyroidism
• Chronic rheumatic heart diseases (obstructive forms such as aortic/pulmonary stenosis	• Hyperparathyroidism
• Chronic lung disease, pulmonary embolism, primary pulmonary hypertension. These affect only the right ventricle and can dilate in untreated cases	• Phaeochromocytoma
• Congenital heart diseases – obstructive types	• Acromegaly
(ii) Dilated	CARDIOFACIAL
• Hypertensive heart disease/failure (of different grades and severity)	• Noonan syndrome
• Alcohol heart disease	• Lentiginosis
• Myocarditis e.g. viral, bacterial, protozoal, rickettsial	NEUROMUSCULAR/NEUROLOGICAL
• Chronic rheumatic heart diseases (regurgitant forms such as mitral/aortic/tricuspid/pulmonary regurgitation	• Friedreich’s ataxia
• Ischaemic cardiomyopathy	• Duchenne-Becker muscular dystrophy
(iii) Associated with pregnancy	• Emery-Dreifuss muscular dystrophy
• Peripartum heart disease	• Myotonic dystrophy
(iv) Fibrotic/obliterative	• Neurofibromatosis
• Löeffler’s endomyocardial disease	• Tuberous sclerosis
• Endomyocardial fibrosis	NUTRITIONAL DEFICIENCIES
(v) Unknown cause	• Beri beri (thiamine)
• Dilated cardiomyopathy	• Pellagra
• Left ventricular non-ischaemic ventricular aneurysms	• Scurvy
	• Selenium
	• Carnitine
	• Kwashiorkor
	AUTOIMMUNE/COLLAGEN
	• Systemic lupus erythematosus
	• Dermatomyositis
	• Rheumatoid arthritis
	• Scleroderma
	• Polyarteritis nodosa
	ELECTROLYTE IMBALANCE
	
	CONSEQUENCE OF CANCER THERAPY
	• Anthracyclines: doxorubicin (adriamycin), daunorubicin
	• Cyclophosphamide
	• Radiation

The classification we are proposing is a modification of that of Maron *et al*.^17^ but we believe that the diseases should be named according to what caused them. All myocardial disorders are included under the classification we are proposing, while less emphasis was placed on the term cardiomyopathy, which, as observed earlier, has now acquired several meanings. It was, however, retained for only those diseases for which the term has become part of the general usage, for example hypertrophic cardiomyopathy. The name dilated cardiomyopathy was used for only those diseases that are characterised by dilatation of the left ventricle with systolic dysfunction, whose cause could not be determined after all the necessary investigations had been performed.
